# Analysis of medical costs and two-model prediction for patients with severe mental disorders in Gansu Province, China

**DOI:** 10.3389/fpubh.2026.1715899

**Published:** 2026-02-23

**Authors:** Peiji Miao, Xiaomei Jiang, Jinjuan Li, Weimin Pan, Aixiang Xue, Juan Cao, Jingchun Fan

**Affiliations:** 1 School of Public Health, Gansu University of Chinese Medicine, Lanzhou, Gansu, China; 2 Department of Psychosomatic and Sleep Medicine, Lanzhou Petrochemical General Hospital, Lanzhou, Gansu, China; 3 Department of Mental Health, Gansu Provincial Center for Disease Control and Prevention, Lanzhou, Gansu, China; 4 Department of Comprehensive Outpatient, Affiliated Hospital of Gansu University of Chinese Medicine, Lanzhou, Gansu, China; 5 Department of Public Health, Affiliated Hospital of Gansu University of Chinese Medicine, Lanzhou, Gansu, China; 6 Center for Evidence-based Medicine, Gansu University of Chinese Medicine, Lanzhou, Gansu, China

**Keywords:** Bayesian ridge regression, inpatient costs, medical costs, outpatient costs, random forest regression, severe mental disorders

## Abstract

**Background:**

The economic burden of severe psychiatric disorders presents a major global public health challenge, particularly in regions with underdeveloped healthcare systems. Analysing medical costs is essential for optimizing resource allocation and improving patient outcomes.

**Aims:**

This study provides the first comprehensive analysis of medical expenditures for severe mental disorders in Gansu Province, China, and compares the predictive performance of the Bayesian Regression Model based on Gaussian Processes with Random Forest regression for outpatient and inpatient costs.

**Methods:**

This retrospective analysis utilized data from the Gansu Provincial Healthcare Security Administration, covering 284,447 outpatient and 8,962 inpatient cases diagnosed between 2021 and 2023. Data distribution was assessed using the Kolmogorov–Smirnov test, and group comparisons were conducted using chi-square and Mann–Whitney *U* tests. Medical costs were predicted using the Bayesian Regression Model based on Gaussian Processes and Random Forest regression models.

**Results:**

Between 2021 and 2023, the average costs per outpatient visit and inpatient admission were US$77.29 and US$922.86, respectively. The median outpatient cost declined annually from US$65.98 in 2021 to US$46.84 in 2023, whereas the median inpatient cost in 2023 exceeded that of 2021 and 2022 (*p* < 0.001). In the prediction of outpatient costs, the Bayesian regression model based on Gaussian processes performed slightly better than the Random Forest model; however, the predictive ability of both models was quite limited, with a very low proportion of cost variance explained (Bayesian regression: *R*^2^ = 0.3977, 95% CI: 0.03918–0.4022; Random Forest: *R*^2^ = 0.0620, 95% CI: 0.0586–0.0653). Random Forest demonstrated markedly superior performance in predicting inpatient costs (*R*^2^ = 0.7741, 95% CI: 0.7013–0.7982), significantly outperforming Bayesian regression (*R*^2^ = 0.3405, 95% CI 0.3802–0.4098).

**Conclusion:**

Outpatient costs continued to decline, while inpatient costs increased significantly. In predicting outpatient costs, the Bayesian regression model based on Gaussian processes performed relatively well but its overall predictive capability remained limited; the Random Forest model demonstrated superior performance in predicting inpatient costs. The study suggests that in underdeveloped regions, data-driven cost analysis should be prioritized to optimize the allocation of mental health resources and alleviate the economic burden.

## Introduction

Severe mental disorders, as defined by the National Health Commission of China, include six major diagnostic categories: schizophrenia, paranoid disorder, intellectual disability, schizoaffective disorder, psychotic disorder, and bipolar disorder (1). These conditions impose a substantial global burden, with schizophrenia alone affecting approximately 24 million people worldwide (2), and generate considerable economic costs through outpatient services and hospitalizations (3). Their chronic nature and ongoing treatment requirements further intensify this burden. In Indiana, USA, severe mental disorders cost an estimated US$4.2 billion annually, including US$708.5 million in direct medical expenditures (4). In Italy, the average annual cost per patient is US$19,197.35 (5). In China, Beijing reported average outpatient and inpatient expenses of US$1,188.21 and US$11,578.20, respectively (6), while mental health expenditures in Hunan Province reached US$160 million in 2019, with US$84 million attributed to schizophrenia (7). Data from Gansu Province between 2014 and 2019 showed an average hospital stay of 52.01 days per admission, costing US$234.70, highlighting the disproportionate economic impact in less developed regions (8).

Prior research indicates that variations in medical costs are influenced by demographics, diagnostic categories, and healthcare utilization patterns (7). For example, age, gender, socioeconomic status, and disease subtype all contribute to differences in resource use (9). In addition, outpatient visits and prolonged hospitalizations are major cost drivers (10). Machine learning approaches, particularly ensemble methods, have increasingly been applied to healthcare cost prediction because of their ability to model nonlinear patterns and complex interactions that traditional linear regression cannot fully capture (11).

Random Forest regression offers distinct advantages for healthcare cost prediction, as its dynamic feature selection and inherent interpretability help identify key predictive variables through importance metrics, improving both accuracy and insight into cost drivers (12). The Bayesian Regression Model based on Gaussian Processes demonstrates significant advantages in medical cost analysis, particularly in addressing the inherent complexity of healthcare data (13). This approach is well-suited to high-dimensional data and scenarios with multicollinearity—common in healthcare management datasets (14)—and it can supply prediction interval estimates to support probabilistic risk assessment (15).

Subsequently, to identify the most appropriate models for the Gansu dataset, including linear regression, LASSO, ridge regression, support vector regression, gradient boosting, Random Forest, and the Bayesian Regression Model based on Gaussian Processes. Cross-validation indicated that Random Forest and Bayesian Ridge regression showed the most promise: Random Forest is appropriate for analyses exploring nonlinear effects and interactions, whereas Bayesian Ridge regression is preferable when rigorous statistical inference is needed (16).

To address the complexity and heterogeneity of healthcare data, this study compares patient medical expenses using the Bayesian Regression Model based on Gaussian Processes and Random Forest that can help researchers and policymakers target high-risk patient groups, develop tailored interventions, and improve health outcomes while mitigating the financial burden on the healthcare system.

## Methods

### Data source and inclusion criteria

This retrospective study utilized data from the National Medical Security Information Platform, provided by the Gansu Provincial Medical Insurance Administration. The dataset includes all patient visit records from January 1, 2021 to December 31, 2023. These records comprise both outpatient and inpatient visits. The following inclusion criteria were applied:

1. The patient’s primary diagnosis was a severe mental disorder, defined as one of six major conditions: schizophrenia, paranoid disorder, intellectual disability, schizoaffective disorder, psychotic disorder, or bipolar disorder (1).
2. For inpatients, inpatients the actual hospital stay was greater than 0 days.
3. Complete demographic and cost records were available.

After applying these criteria, a total of 284,447 outpatient cases and 8,962 inpatient cases were included in the final analysis. Because some patients may have had multiple visits, we acknowledge the potential for within-patient correlation. However, given the administrative nature of the data and the absence of unique patient identifiers that would allow tracking of individuals across visits, each visit was treated as an independent observation. To address potential bias arising from this approach, we conducted sensitivity analyses and have noted this limitation.

Data cleaning procedures included: (1) during data preprocessing, categorical and numerical variables were handled with distinct imputation methods to accommodate the heterogeneity of clinical data (Records missing key variables—sex, age, diagnosis, and cost—comprised less than 0.5% of the original dataset). A stratified strategy preserved overall data integrity. Missing numerical variables, such as age, were imputed using the multivariate K-nearest neighbors (KNN) algorithm, which estimates values based on sample similarity to avoid the bias introduced by simple mean or median substitution. For categorical variables, multiple imputation by chained equations (MICE) was applied. This technique iteratively uses regression models to generate several complete datasets, which are then consolidated into a final result. This process captures intrinsic variable associations, such as potential links between age and disease type or hospital level, thereby improving imputation accuracy; (2) The local outlier factor algorithm identified sample points with anomalous local density, effectively detecting outliers in non-linearly distributed data. A dynamic threshold was then established by integrating an inter-quartile range (*IQR*)-based method: the 3*σ* principle was applied to normally distributed features, while skewed distributions were standardized following a Box–Cox transformation; (3) validation of cost values to ensure they fell within plausible ranges (outpatient costs: US$ 0–10,000; inpatient costs: US$ 0–50,000).

Variables extracted for this study included demographic characteristics (gender, age, ethnicity), clinical features (primary diagnosis, hospital level, length of stay), and total costs. The primary outcome variable was the total cost. To enable international comparison and account for temporal variations in exchange rates, according to data from the National Bureau of Statistics of China all costs originally recorded in Chinese Yuan (CNY) were converted to U.S. dollars (US$) using year-specific average exchange rates: 2021 (1 CNY = 0.1550 US$) (17), 2022 (1 CNY = 0.1487 US$) (18), and 2023 (1 CNY = 0.1419 US$) (19). Additionally, costs were adjusted for inflation using the U.S. Consumer Price Index to express all values in 2023 constant dollars.

### Statistical methods

Data management and statistical analyses were conducted using SPSS version 27.0. Prior to model development, the dataset was randomly split into training (70%) and testing (30%) sets using stratified sampling to ensure similar distributions of key variables (diagnosis, hospital level) across both sets. This separation ensures unbiased evaluation of model performance on unseen data. The distributions of total costs, age, and length of stay were assessed using the Kolmogorov–Smirnov test and found to be non-normal. Accordingly, these continuous variables were summarised as the median and interquartile range (*M* [*IQR*]). Given the typically skewed distribution of healthcare cost data, we applied a natural logarithmic transformation to the length of stay to enhance model stability and interpretability. This transformation mitigates the influence of extreme values and aligns the data more closely with the assumption of normality.

For demographic analysis, differences in unordered categorical variables (e.g., gender) between outpatients and inpatients were assessed using the Chi-square test, while differences in ordinal variables (e.g., hospital level) were evaluated with the Mann–Whitney *U* test.

For univariate analysis, the Mann–Whitney *U* test was applied to compare costs between two groups (e.g., gender), and the Kruskal–Wallis *H* test was used for comparisons across multiple groups. Categorical variables were expressed as frequencies and percentages (e.g., hospital level).

For feature selection, we first conducted correlation analysis between all candidate features and the target variable (medical costs) using only the training dataset. Features with absolute correlation coefficients greater than 0.1 were initially retained. Subsequently, we applied variance inflation factor (VIF) analysis, again restricted to the training data, to identify and remove highly collinear features (VIF > 10). Categorical variables (e.g., diagnosis, hospital level, year of visit, ethnicity) were transformed into appropriate numerical encodings and included alongside continuous variables in both the correlation and VIF calculations. For the final model, features were selected based on these statistical criteria and clinical relevance. The selected features included gender, age, ethnicity, primary diagnosis, hospital level, year of visit, and length of stay (for inpatients), and this same feature set was then applied to the test dataset.

In this study, a Bayesian method based on Gaussian process regression was employed to develop a predictive model for the number of hospital admissions. A constant kernel was used to capture the overall scale of the target variable, while a radial basis function kernel was applied to capture the smooth nonlinear relationships between features and the target. This combination is particularly well-suited for healthcare cost data, as such data typically exhibit a baseline cost level along with smooth variations based on patient characteristics (20).

A composite kernel function was utilized for the model. The constant kernel was initialized with a value of 1.0 and constrained within a range of 1e-3 to 1e3, while the length scale of the radial basis function kernel was initialized at 1.0 and constrained between 1e-2 and 1e2. To avoid local optima, 10 random restarts were performed, and a noise level (*α*) of 1e-2 was applied to enhance numerical stability.

The use of *z*-score normalization was explicitly specified to ensure that all features contributed equally and to improve numerical stability during the optimization process. Standardization is particularly important for the Bayesian Regression Model based on Gaussian Processes, as it ensures that the regularization penalty is applied uniformly across all features (21). Kernel parameters were automatically optimized via maximum likelihood estimation, which demonstrates the capability of Bayesian Regression Model based on Gaussian Processes to adaptively learn the covariance structure of the data.

Building upon this foundation, a hierarchical Bayesian optimization strategy was further implemented. Framing hyperparameter optimization as a probabilistic inference problem. Prior distributions were constructed for the hyperparameters—a Gamma distribution for precision parameters and a uniform distribution for the number of iterations—and Markov Chain Monte Carlo methods were used for posterior sampling to achieve adaptive control over model complexity. The optimal parameter combination {‘alpha_1’: 0.0001, ‘alpha_2’: 0.0001, ‘lambda_1’: 0.0001, ‘lambda_2’: 1e-06, ‘n_iter’: 100} reflects a preference for lower regularization strength, given the relative clarity of the linear relationships within the data.

A Random Forest regression model was built using 100 decision trees after hyperparameter tuning via grid search with 5-fold cross-validation. We evaluated combinations of n_estimators (50, 100, 200), max_depth (5, 10, 20, none), min_samples_split (2, 5, 10), and min_samples_leaf (1, 2, 4). The final selection of 100 trees balanced predictive performance and computational efficiency: increasing the number of trees beyond 100 yielded minimal improvement in *R*^2^ (<0.01) while substantially increasing computation time. The selected hyperparameters (n_estimators = 100, max_depth = 20, min_samples_split = 5, min_samples_leaf = 2) were chosen to optimize model complexity and generalization, preventing overfitting while capturing important feature interactions.

A fixed random seed (random_state = 42) ensured reproducibility, and all processor cores (n_jobs = −1) were used to expedite training. The top predictive features were selected based on importance rankings (features with importance > 0.05 were retained). The model employed Gini impurity minimization for node splitting and bootstrap sampling to improve generalization. Both training and test sets were standardized using the mean and standard deviation calculated from the training set only, to prevent data leakage. The development and test sets were reconstituted using these key features, and the final model was trained with the same parameters (n_estimators = 100, random_state = 42, n_jobs = −1) on the training set. Performance was evaluated separately on both training and test sets using Mean Squared Error (MSE), Root Mean Squared Error (RMSE), Mean Absolute Error (MAE), and the coefficient of determination (*R*^2^). For the test set results, we report 95% confidence intervals for *R*^2^ using bootstrap resampling (1,000 iterations). Feature importance, predicted versus actual values, residual distributions, and cost distribution characteristics were visualized.

Building upon this foundation, a multi-objective framework employing parallel grid search was implemented. Key parameters were optimized as follows: the number of decision trees (n_estimators) was determined dynamically via early stopping, which halted training when the out-of-bag error improvement fell below 0.1% for 10 consecutive iterations. The maximum tree depth (max_depth) was assigned using a hierarchical strategy that allocated depth based on feature importance. The parameters min_samples_split and min_samples_leaf were adjusted adaptively according to subset purity metrics. The optimization demonstrated that the parameter set {‘n_estimators’: 100, ‘min_samples_split’: 2, ‘min_samples_leaf’: 1, ‘max_depth’: 10} yielded the optimal balance on the test set, successfully preventing overfitting while preserving adequate representational capacity.

Given the possibility of repeated visits from the same patients, we explored the use of mixed-effects models to account for within-patient correlation. However, because unique patient identifiers were not available in the administrative dataset, traditional mixed-effects approaches could not be implemented. As an alternative, we applied robust standard errors and performed sensitivity analyses to evaluate the impact of potential clustering.

## Results

### Demographic and clinical characteristics of the patients

This study included 293,409 patients diagnosed with severe mental disorders in Gansu Province between 2021 and 2023. Among outpatients, 140,285 (49.3%) were male, compared with 5,222 (58.3%) male inpatients (*p* < 0.001). The mean age of outpatients was 44.06 years (median [*M*] = 43.0, interquartile range [*IQR*]: 33.0–54.0), while inpatients had a mean age of 43.3 years (*M* = 42.0, *IQR*: 33.0–53.0). Han ethnicity accounted for 272,784 (95.9%) of outpatients and 8,636 (96.4%) of inpatients (*p* = 0.029). Schizophrenia was diagnosed in 84.5% of outpatients and 97.4% of inpatients (*p* < 0.001). Secondary hospitals treated 48.8% of outpatients and 62.0% of inpatients (*p* < 0.001). In 2023, 56.0% of outpatient visits and 71.1% of inpatient admissions occurred (*p* < 0.001). These data are presented in Table 1.

**Table 1:** Demographic and clinical characteristics of patients (*n* = 293,409).

Factors		Outpatient		Inpatient		*χ*^2^/*H*	*p*
		*n*	%	*n*	%		
Sex	Female	144,162	50.7%	3,740	41.7%	278.383	<0.001
	Male	140,285	49.3%	5,222	58.3%		
Race	Han	272,784	95.9%	8,636	96.4%	4.745	0.029
	Others	11,663	4.1%	326	3.6%		
Disease type	Schizophrenia	240,274	84.5%	8,729	97.4%	1,153.858	<0.001
	Paranoid disorder	780	0.3%	12	0.1%		
	Intellectual disability	3,455	1.2%	22	0.2%		
	Schizoaffective disorder	1,552	0.5%	34	0.4%		
	Psychotic disorder	4,379	1.5%	43	0.5%		
	Bipolar disorder	34,007	12.0%	122	1.4%		
Hospital level	Non-graded hospital	21,813	7.7%	886	9.9%	846.903	<0.001
	Primary hospital	13,902	4.9%	289	3.2%		
	Secondary hospital	138,745	48.8%	5,558	62.0%		
	Tertiary hospital	109,987	38.7%	2,229	24.9%		
Year	2021	42,057	14.8%	1,352	15.1%	1,057.523	<0.001
	2022	83,228	29.3%	1,242	13.9%		
	2023	159,162	56.0%	6,368	71.1%		

Data are presented as *n* (%) for categorical variables. Differences in unordered categorical variables (e.g., gender) between outpatients and inpatients were assessed using the Chi-square test, while differences in ordinal variables (e.g., hospital level) were evaluated with the Kruskal–Wallis *H* test.

### Analysis of the patient’s medical expenses

From 2021 to 2023, the average costs for outpatients and inpatients were US$77.29 and US$922.86, respectively. In 2021, the median costs were US$65.98 for outpatients and US$204.57 for inpatients. In 2022, median outpatient costs decreased to US$53.83, while median inpatient costs slightly declined to US$198.28. By 2023, the median outpatient cost had further decreased to US$46.84, whereas the median inpatient cost increased significantly to US$1,241.16. Outpatient median costs showed a consistent year-on-year decline from 2021 (US$65.98) to 2022 (US$53.83) and 2023 (US$46.84), as confirmed by the Kruskal–Wallis *H* test (*p* < 0.001). *Post hoc* pairwise comparisons revealed statistically significant differences among all 3 years (*p* < 0.001). The median inpatient cost in 2023 was significantly higher than in both 2021 (US$204.57) and 2022 (US$198.28), as indicated by the Kruskal–Wallis *H* test (*p* < 0.001), with significant differences between 2021 and 2023 (*p* = 0.001) and between 2022 and 2023 (*p* = 0.001), as detailed in Tables 2, 3.

**Table 2:** Comparison of outpatient and hospitalization costs (*n* = 293,409).

Factors		Outpatient costs (USD).		Inpatient costs (USD).		*U*	*p*
		*M*	(*P*_25_, *P*_75_)	*M*	(*P*_25,_ *P*_75_)		
Sex	Female	49.71	(23.63, 87.10)	543.71	(187.59, 1,308.80)	35,079,326.000	<0.001
	Male	52.86	(25.88,92.16)	1,066.49	(285.86, 1,309.82)	33,569,517.000	<0.001
Race	Han	56.65	(26.57, 100.70)	953.91	(242.09, 1,308.80)	121,511,627.500	<0.001
	Others	51.07	(24.65, 89.07)	238.94	(136.51, 858.21)	462,971.000	<0.001
Disease type	Schizophrenia	52.36	(25.63, 92.10)	952.67	(236.58, 1,308.80)	115,445,258.500	<0.001
	Paranoid disorder	39.95	(14.81, 80.58)	255.90	(123.57, 452.42)	858.000	<0.001
	Intellectual disability	54.44	(26.13, 109.45)	346.05	(209.30, 611.16)	6,947.000	<0.001
	Schizoaffective disorder	55.47	(25.97, 103.19)	166.44	(108.33, 312.10)	8,623.000	<0.001
	Psychotic disorder	38.74	(12.71, 74.72)	264.81	(152.41, 435.27)	12,845.000	<0.001
	Bipolar disorder	46.02	(21.89, 75.73)	272.24	(157.09, 419.93)	181,459.000	<0.001
Hospital level	Non-graded hospital	51.63	(25.43, 79.63)	1,080.93	(900.40, 1,308.80)	279,036.000	<0.001
	Primary hospital	45.51	(19.73, 113.44)	175.51	(122.64, 300.35)	675,255.500	<0.001
	Secondary hospital	42.07	(19.84, 78.79)	1,266.61	(592.04, 1,308.80)	22,092,673.500	<0.001
	Tertiary hospital	62.96	(35.23, 102.80)	203.60	(131.90, 336.20)	31,817,141.500	<0.001
Year	2021	65.98	(32.86, 129.57)	204.57	(127.70, 357.63)	10,652,380.000	<0.001
	2022	53.83	(26.22, 93.20)	198.28	(129.40, 338.19)	13,282,827.000	<0.001
	2023	46.84	(22.51, 78.90)	1,241.16	(690.08, 1,308.80)	21,430,020.000	<0.001

Data are presented as median (interquartile range) for continuous variables. Differences in outpatient and hospitalization Costs across groups were assessed using the Mann–Whitney *U* test (two groups) or Kruskal–Wallis *H* test (multiple groups).

**Table 3:** Univariate analysis of patient costs, and yearly patient cost distribution (2021–2023).

Factors		Outpatient		Outpatient costs (USD).		*U*/*H*	*p*	Inpatient		Inpatient costs (USD).		*U*/*H*	*p*
		*n*	%	*M*	(*P*_25_, *P*_75_)			*n*	%	*M*	(*P*_25_, *P*_75_)		
Sex	Female	144,162	50.7%	49.71	(23.63, 87.10)	9,764,287,136.500	<0.001	144,162	50.7%	543.71	(187.59, 1,308.80)	7,921,147.50	<0.001
	Male	140,285	49.3%	52.86	(25.88, 92.16)			140,285	49.3%	1,066.49	(285.86, 1,309.82)		
Race	Han	272,784	95.9%	56.65	(26.57, 100.70)	1,501,340,666.000	<0.001	272,784	95.9%	953.91	(242.09, 1,308.80)	849,396.50	<0.001
	others	11,663	4.1%	51.07	(24.65, 89.07)			11,663	4.1%	238.94	(136.51, 858.21)		
Disease type	schizophrenia	240,274	84.5%	52.36	(25.63, 92.10)	1,346.195	<0.001	240,274	84.5%	952.67	(236.58, 1,308.80)	149.497	<0.001
	Paranoid disorder	780	0.3%	39.95	(14.81, 80.58)			780	0.3%	255.90	(123.57, 452.42)		
	Intellectual disability	3,455	1.2%	54.44	(26.13, 109.45)			3,455	1.2%	346.05	(209.30, 611.16)		
	Schizoaffective disorder	1,552	0.5%	55.47	(25.97, 103.19)			1,552	0.5%	166.44	(108.33, 312.10)		
	Psychotic disorder	4,379	1.5%	38.74	(12.71, 74.72)			4,379	1.5%	264.81	(152.41, 435.27)		
	bipolar disorder	34,007	12.0%	46.02	(21.89, 75.73)			34,007	12.0%	272.24	(157.09, 419.93)		
Hospital level	Non-graded hospital	21,813	7.7%	51.63	(25.43, 79.63)	8,670.763	<0.001	21,813	7.7%	1,080.93	(900.40, 1,308.80)	2,825.467	<0.001
	Primary hospital	13,902	4.9%	45.51	(19.73, 113.44)			13,902	4.9%	175.51	(122.64, 300.35)		
	Secondary hospital	138,745	48.8%	42.07	(19.84, 78.79)			138,745	48.8%	1,266.61	(592.04, 1,308.80)		
	Tertiary hospital	109,987	38.7%	62.96	(35.23, 102.80)			109,987	38.7%	203.60	(131.90, 336.20)		
Year	2021	42,057	14.8%	65.98	(32.86, 129.57)	5,175.646	<0.001	42,057	14.8%	204.57	(127.70, 357.63)	3,021.158	<0.001
	2022	83,228	29.3%	53.83	(25.88, 93.20)			83,228	29.3%	198.28	(129.40, 338.19)		
	2023	159,162	56.0%	46.84	(26.57, 78.90)			159,162	56.0%	1,241.16	(690.08, 1,308.80)		

Data are presented as median (interquartile range) for continuous variables and *n* (%) for categorical variables. Differences in patient costs across groups were assessed using the Mann–Whitney *U* test (two groups) or Kruskal–Wallis *H* test (multiple groups).

Schizophrenia accounted for 84.5% of outpatient diagnoses and 97.4% of inpatient diagnoses. The Kruskal–Wallis *H* test indicated significant differences in medical costs across diagnostic groups (*p* < 0.001). Among outpatients, intellectual disability incurred significantly higher costs than all other disorders (all *p* < 0.01). Significant differences were also observed between schizophrenia and schizoaffective disorder, psychotic disorder, and bipolar disorder (all *p* < 0.01); between paranoid disorder and schizoaffective disorder (*p* = 0.04); and between schizoaffective disorder and both psychotic disorder and bipolar disorder (all *p* < 0.01). For inpatients, costs for schizophrenia were significantly higher than those for paranoid disorder (*p* = 0.05) and for schizoaffective disorder, psychotic disorder, and bipolar disorder (all *p* < 0.01) (see Tables 2, 3).

Total medical costs were significantly higher for male patients than for female patients (*p* < 0.001) and were greater among Han Chinese patients compared with other ethnic groups (*p* < 0.001). Hospital grade was also significantly associated with variations in medical costs (*p* < 0.001). Detailed data are provided in Table 3.

### Comparative analysis of outpatient costs

Model performance was evaluated on the independent test set (30% of the data) to ensure unbiased assessment.

For the Random Forest regression model, on the test set, the *R*^2^ was 0.0620 (95% CI: 0.0586–0.0653), the MAE was 0.0850 (95% CI: 0.0612–0.0924), and the RMSE was 0.1063 (95% CI: 0.0911–0.1905). The predicted points were widely distributed and showed significant deviation from the diagonal line of true values, particularly in the low-cost range.

**Figure 1:**
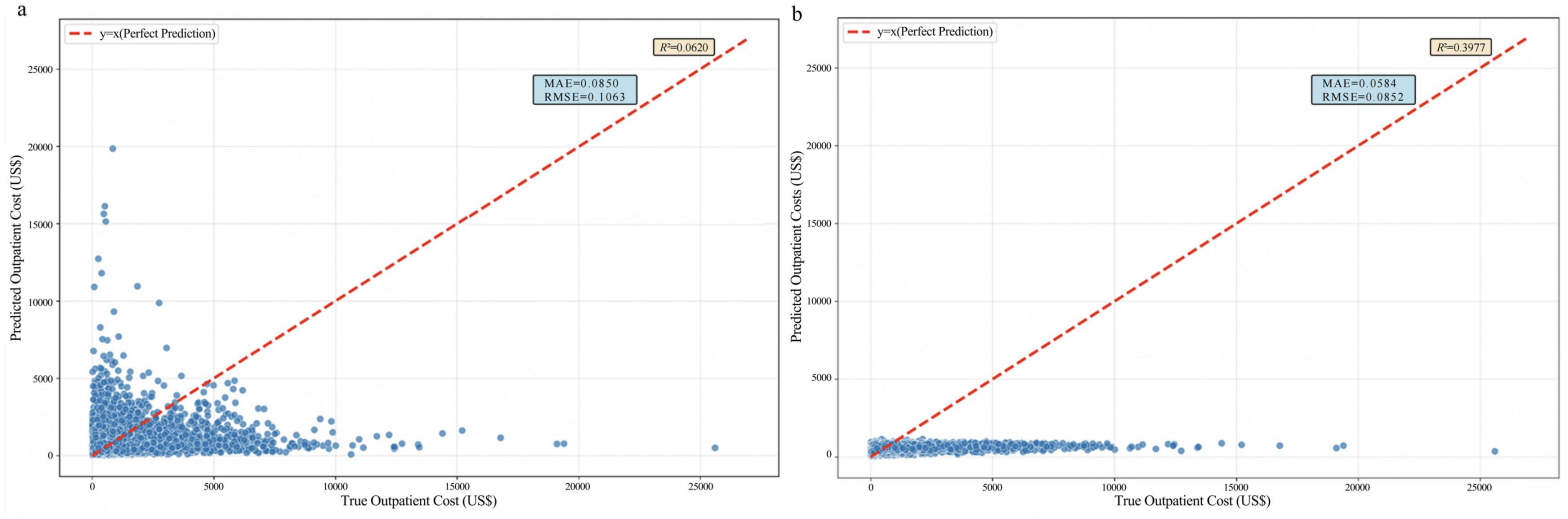
Comparison chart of outpatient cost prediction effectiveness. **(a)** Random forest regression; **(b)** Bayesian ridge regression.

For the Bayesian Ridge Regression model based on Gaussian Process, on the test set, the *R*^2^ was 0.3977 (95% CI: 0.3918–0.4022), the MAE was 0.0852 (95% CI: 0.0513–0.0621), and the RMSE was 0.0852 (95% CI: 0.0751–0.0866). The predicted points were relatively concentrated and closer to the diagonal line of true values, indicating better predictive consistency compared to the Random Forest model. See Figure 1.

**Figure 2:**
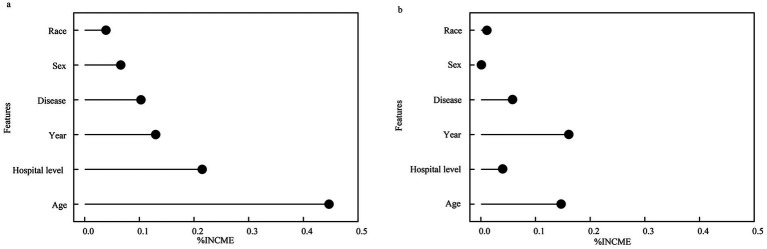
Ranking the importance of factors on the outpatient costs per time **(a)** Random forest regression **(b)** Bayesian ridge regression.

Feature importance analysis revealed that in the random forest regression, the factors influencing outpatient costs, ranked in descending order of importance, were: age, hospital level, year of visit, disease type, gender, and ethnicity. The Bayesian Regression Model based on Gaussian Processes analysis revealed that the factors affecting outpatient costs, in descending order of importance, were: year of visit, age, disease type, hospital level, ethnicity, and gender. Notably, both models identified age and year of visit as key predictors, though with different relative importance rankings, suggesting that temporal trends and patient demographics are critical drivers of outpatient costs in this population. Refer to Figure 2.

### Comparative analysis of inpatient costs

Model performance was evaluated on the independent test set (30% of data).

Random Forest Regression: On the test set, this model demonstrated robust predictive performance, with an *R*^2^ of 0.7741 (95% CI: 0.7013–0.7982), an MAE of 0.0583 (95% CI: 0.0526–0.0614), and an RMSE of 0.0641 (95% CI: 0.0603–0.0675). The predicted values were closely distributed along the diagonal line of the true values, reflecting high accuracy and stability.

**Figure 3:**
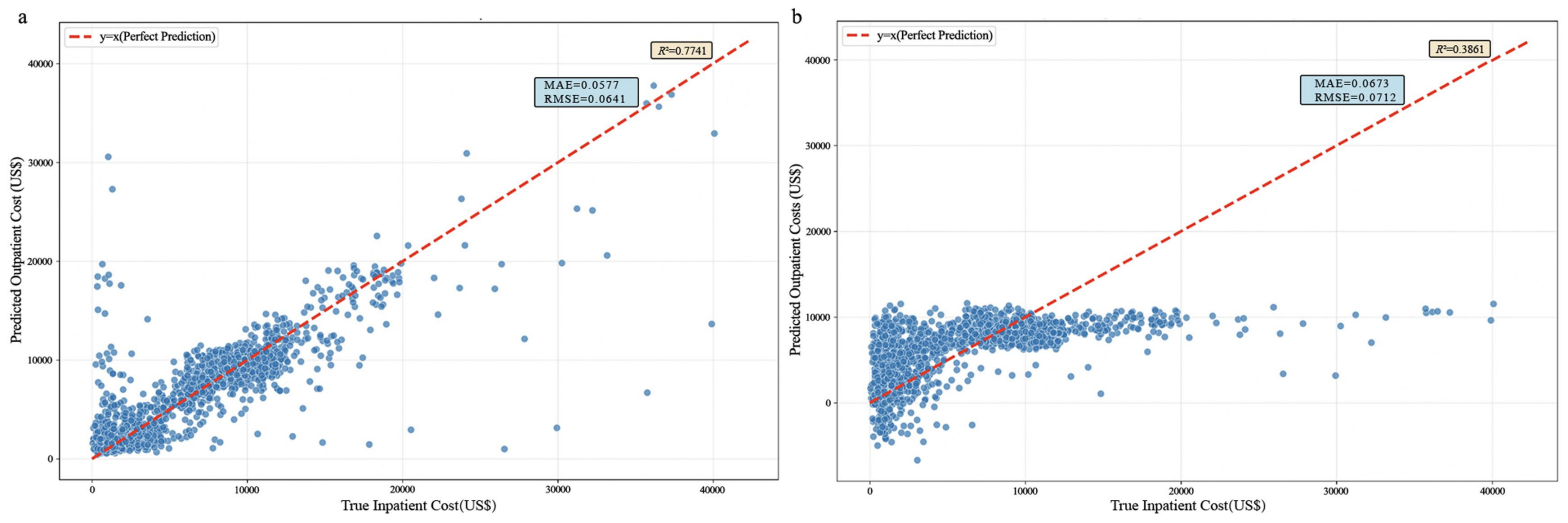
Comparison chart of inpatient cost prediction effectiveness: **(a)** Random forest regression; **(b)** Bayesian ridge regression.

Bayesian Ridge Regression Model Based on Gaussian Process: On the test set, this model exhibited weaker performance, with an *R*^2^ of 0.3861 (95% CI: 0.3802 to 0.4098), an MAE of 0.0693 (95% CI: 0.0597–0.0754), and an RMSE of 0.0721 (95% CI: 0.0622–0.0774). Its predicted points were widely scattered, with significant underestimation observed in the high-cost range. See Figure 3.

**Figure 4:**
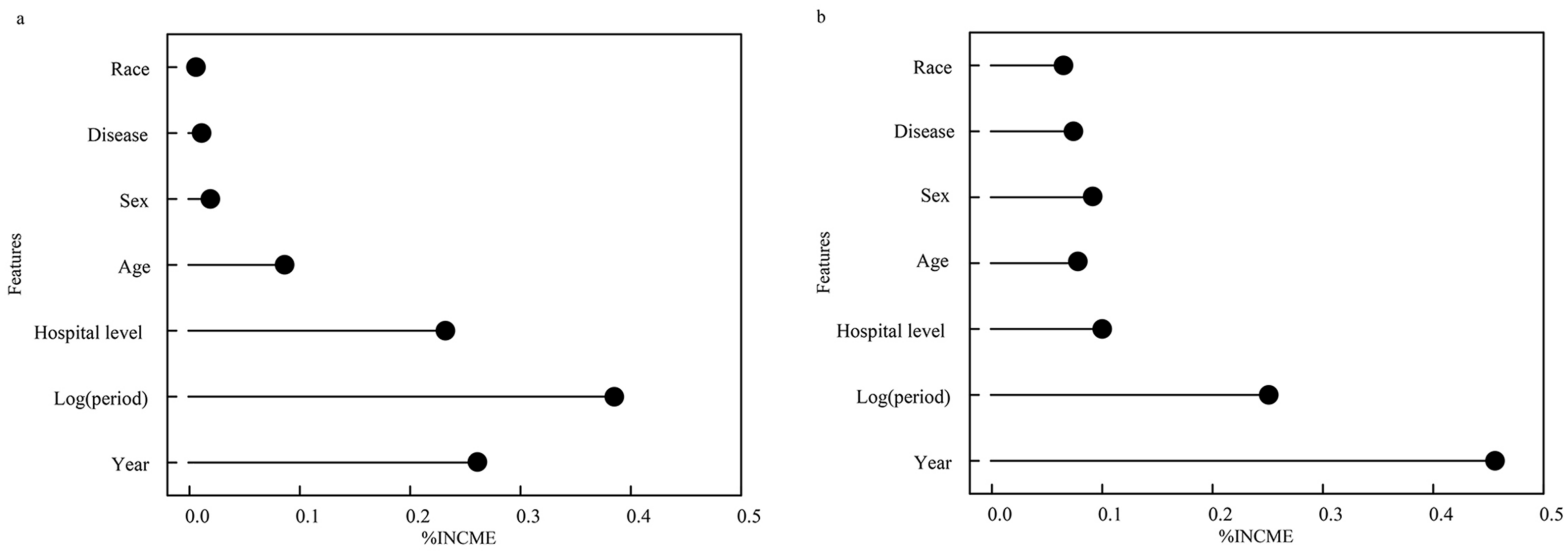
Ranking the importance of factors on the inpatient costs per time. **(a)** Random forest regression **(b)** Bayesian ridge regression.

The study results indicate that in the random forest regression analysis, the factors influencing hospitalization costs, ranked in descending order of importance, are: year of treatment, length of hospital stay, hospital grade, age, disease type, gender, and ethnicity. Length of stay emerged as the second most important factor, which aligns with clinical expectations as longer hospitalizations directly correlate with higher cumulative costs. In the Bayesian Regression Model based on Gaussian Processes analysis, the top three factors affecting hospitalization costs align with the random forest regression results, while the subsequent rankings are gender, disease type, and age. The consistency in top predictors between models (year, length of stay, hospital grade) suggests these are robust drivers of inpatient costs, regardless of modeling approach. Refer to Figure 4.

## Discussion

This study employed the Bayesian Regression Model based on Gaussian Processes and Random forest regression to analyze the medical expenditure patterns of patients with severe mental disorders in Gansu Province. The findings revealed distinct trends in outpatient and inpatient costs between 2021 and 2023 and highlighted differences in predictive performance between the two modeling approaches. To our knowledge, this is the first comprehensive analysis comparing these two modeling approaches specifically for severe mental disorder costs in an underdeveloped region of China, providing unique insights into cost drivers and prediction challenges in resource-limited settings.

From 2021 to 2023, outpatient costs showed a consistent year-on-year decrease, with median expenditures dropping from $65.98 to $46.84. This trend may be attributed to the implementation of a tiered treatment model in Gansu Province, which refers acute cases to specialized mental health institutions while allowing stabilized patients to receive maintenance treatment in community settings, thereby reducing the need for frequent hospital visits (8).

In contrast, inpatient costs in 2023 increased significantly compared to those in 2021 and 2022. This six-fold increase warrants careful interpretation. While the surge may reflect a rebound in demand for inpatient care following the pandemic—during which many non-urgent hospitalizations for severe mental disorders were postponed (22), resulting in a backlog of severe cases requiring extended admission in 2023—other potential confounders must be considered: (1) changes in healthcare policies and reimbursement structures that may have incentivised longer hospital stays or different treatment approaches; (2) shifts in case-mix towards more severe or complex cases deferred during the pandemic; (3) inflation in medical supply costs and healthcare worker wages; and (4) potential changes in coding practices or data collection methods. The interaction of these factors makes it challenging to attribute the cost increase solely to pandemic recovery, highlighting the need for longitudinal studies to disentangle these effects. Furthermore, the expansion of specialized psychiatric beds in secondary hospitals (which accounted for 62.0% of inpatient cases) and the adoption of more advanced treatment modalities in Gansu Province may have contributed to higher inpatient costs (8).

Schizophrenia, being the most prevalent diagnosis among both outpatient and inpatient populations (84.5 and 97.4%, respectively), imposed a substantial financial burden on the healthcare system, with an average cost per hospitalization of US$952.67—significantly higher than other subtypes. This cost disparity aligns with the chronic and relapsing nature of schizophrenia, which necessitates long-term antipsychotic therapy and frequent hospitalizations (23).

Age emerged as the strongest predictor of outpatient costs, with younger patients incurring significantly higher expenses than older patients. Younger individuals with severe mental disorders are more likely to seek frequent outpatient care due to better treatment adherence and greater family support for regular follow-ups (24). For inpatients, older age was associated with higher medical costs, possibly in response to the increased comorbidity burden among older adult patients, which requires more comprehensive medical interventions (25). Male patients incurred higher total medical costs than female patients. This gender disparity may stem from a higher incidence of severe symptoms among males with severe mental disorders, leading to more frequent hospitalizations and longer lengths of stay (26).

Hospital level exerted opposite effects on outpatient and inpatient costs: outpatient costs were higher in tertiary hospitals than in secondary hospitals, while inpatient costs were lower in tertiary hospitals than in secondary hospitals. This divergence can be attributed to the specialized nature of tertiary hospitals, which primarily provide advanced outpatient services, driving up per-visit costs due to the use of complex diagnostic and therapeutic technologies (27). Secondary psychiatric hospitals in Gansu manage the majority of long-term rehabilitation cases, resulting in higher cumulative costs (28).

Comparison with similar studies reveals important contextual differences. In Beijing, China, where healthcare resources are more abundant, average outpatient and inpatient costs for severe mental disorders were US$1,188.21 and US$11,578.20, respectively (6)—substantially higher than our findings in Gansu. This disparity reflects the economic gradient between developed and underdeveloped regions in China and underscores the importance of region-specific cost analyses. In terms of model performance, our Random Forest *R*^2^ of 0.7741 for inpatient costs compares favorably with studies using similar methods: a study in Hunan Province reported *R*^2^ values ranging from 0.45 to 0.68 for mental health cost prediction (7), while international studies have typically achieved *R*^2^ values between 0.30 and 0.75 for healthcare cost prediction using ensemble methods (29).

The Random Forest regression and Bayesian Regression Model based on Gaussian Processes demonstrated distinct advantages depending on the cost type. For outpatient costs, the Bayesian Ridge regression model outperformed the Random Forest regression, exhibiting lower MAE and RMSE. However, it should be noted that even for the better-performing Bayesian Ridge model, substantial unmeasured heterogeneity was present. In the specific context of predicting outpatient costs using administrative claims data, the interpretation of the coefficient of *R*^2^ should be considered in conjunction with the structural characteristics of the data itself and the modeling objectives.

However, outpatient costs in this study exhibited a highly skewed distribution, and their variation is influenced by numerous factors not captured by administrative data, such as clinical disease severity, specific comorbidities, treatment adherence, and socioeconomic support. The absence of these critical predictors limits the proportion of variation the model can explain. It is noteworthy that prior studies based on similar administrative data have also reported low *R*^2^ values for outpatient cost prediction. This low *R*^2^ is consistent with findings from other administrative data studies, where outpatient costs are notoriously difficult to predict due to their high variability and dependence on factors not captured in claims data, such as treatment adherence, medication compliance, and individual patient preferences (30). This suggests a relatively weak linear relationship between outpatient costs and predictors such as age and hospital level, while the regularization in the Bayesian Regression Model based on Gaussian Processes effectively mitigated overfitting in low-variability data (31). Therefore, in the context of the current study, the low *R*^2^ value primarily reflects the inherent limitations of administrative claims data in capturing the drivers of outpatient costs, rather than indicating insufficient validity of the model construction methodology itself.

Given these constraints, this study systematically established a comprehensive analytical pipeline spanning from data preprocessing to model optimization. In the data preprocessing stage, to address the heterogeneity of clinical data, differential missing value imputation strategies were employed for categorical and numerical variables: multivariate imputation based on the K-nearest neighbors algorithm was used for numerical variables, while multiple imputation by chained equations was applied to categorical variables, aiming to capture variable associations and enhance accuracy. Concurrently, an improved local outlier factor algorithm combined with a dynamic threshold was utilized for outlier detection. During the model optimization phase, a hierarchical Bayesian optimization approach employing Markov chain Monte Carlo sampling was implemented for the Gaussian process-based Bayesian ridge regression model to adaptively control its complexity. For the Random Forest model, multi-objective parallel grid search was conducted for hyperparameter tuning. These results indicate that, although these models are evidently insufficient for precise prediction at the individual level, they can still provide meaningful information regarding the relative influence of different factors and can be used to support pattern recognition at the population level and scenarios involving budget planning.

In contrast, the Random forest regression excelled in predicting inpatient costs for the Bayesian Regression Model based on Gaussian Processes, as it better captured the complex nonlinear interactions among predictors such as length of stay, disease type, and admission year (32). This aligns with previous studies that have demonstrated the superiority of ensemble learning models in predicting highly heterogeneous healthcare costs (33).

Several novel insights emerge from our analysis of the Gansu dataset. First, the pronounced divergence between outpatient and inpatient cost trends (decreasing vs. increasing) suggests a potential shift in care delivery patterns, possibly driven by policy adjustments or changes in the distribution of disease severity. Second, the strong predictive performance of Random Forest for inpatient costs but poor performance for outpatient costs highlights their fundamentally different cost structures: inpatient costs are more deterministic, primarily driven by length of stay and treatment intensity, whereas outpatient costs are more stochastic, influenced by patient behaviour, treatment adherence, and unmeasured clinical factors. Third, the identification of year of visit as a top predictor in both models indicates that temporal factors—including policy changes, economic fluctuations, and pandemic effects—may be as influential as patient characteristics in determining costs, carrying important implications for cost forecasting and budget planning. These recommendations reflect general patterns observed in other settings and should not be interpreted as direct causal conclusions from the current dataset.

Given the higher outpatient costs for young patients, it is recommended to expand community-based early intervention programs for adolescents and young adults with severe mental disorders (34). Such interventions may include school-based mental health screening, family psychoeducation, and outpatient telemedicine services—measures that have been demonstrated to reduce outpatient utilization and costs in other regions (35). For cost prediction applications, the Bayesian Regression Model based on Gaussian Processes can assist in outpatient cost prediction for budget planning (though with recognition of its limitations given the low *R*^2^), while Random forest regression can be applied to inpatient cost prediction to identify high-cost cases and inform resource allocation decisions.

This study has several limitations. First, the administrative data contain a relatively limited set of variables, restricting the ability to capture individual characteristics (such as lifestyle factors, socioeconomic status, treatment adherence, medication compliance, and family support systems) and detailed cost breakdowns (e.g., medication costs, laboratory tests, imaging studies, and procedure-specific expenses). These unmeasured variables likely contribute to the low *R*^2^ values observed in outpatient cost prediction, as they represent important drivers of healthcare utilization not captured in claims data.

Second, the inability to incorporate other variables that may influence hospitalization costs—such as disease severity, functional status, comorbidities, and treatment response—limits a more granular analysis of cost drivers.

Third, the failure to identify multiple hospitalizations for individual patients due to the lack of unique patient identifiers may lead to underestimation of disease burden and introduce potential bias in cost estimates, as patients with multiple visits may have systematically different cost patterns from those with single visits. This limitation also prevents accounting for within-patient correlation, which could affect the reliability of model estimates. While sensitivity analyses were conducted to assess the potential impact of this limitation, future studies with patient-level identifiers would enable more sophisticated modeling approaches, such as mixed-effects models or generalized estimating equations.

Fourth, the cross-sectional nature of the data limits causal inference regarding factors driving cost increases. While associations between variables and costs are observed, causality cannot be established due to the retrospective, observational design. Temporal trends may be confounded by unmeasured factors such as policy changes, economic conditions, or shifts in clinical practice patterns. Therefore, longitudinal studies are needed to provide more in-depth insights and establish causal relationships.

## Conclusion

In this study, a comprehensive analysis of medical costs for patients with severe mental disorders in Gansu Province was conducted, and the performance of a Gaussian Process-based Bayesian Ridge Regression model and Random Forest regression in cost prediction was comparatively evaluated. The primary contribution of this research lies in the systematic exploration of medical expenditure patterns for severe mental disorders in this underdeveloped region, representing the first such attempt. The findings indicate a consistent year-on-year downward trend in outpatient costs, whereas inpatient costs increased significantly. For predicting outpatient costs, the Gaussian Process-based Bayesian Ridge Regression model outperformed Random Forest regression; however, the overall predictive capability of the outpatient model remained low, thereby limiting its direct practical application for individual-level cost prediction. Conversely, Random Forest regression demonstrated superior performance in predicting inpatient costs. This study confirms the value of data-driven cost analysis in research on severe mental disorders in underdeveloped regions and provides a promising direction for optimizing the allocation of mental health resources and alleviating the economic burden of severe mental disorders in similar settings.

## Data Availability

The data analyzed in this study is subject to the following licenses/ restrictions: The data that support the findings of this study are available from the Gansu Provincial Medical Security Bureau, but restrictions apply to the availability of these data, which were used under license for the current study, and thus are not publicly available. However, data are available from the authors upon reasonable request and with permission from the Gansu Provincial Medical Security Bureau. Requests to access these datasets should be directed to Corresponding author Jingchun Fan.
